# Down regulation of Cathepsin W is associated with poor prognosis in pancreatic cancer

**DOI:** 10.1038/s41598-023-42928-y

**Published:** 2023-10-04

**Authors:** Fatemeh Khojasteh-Leylakoohi, Reza Mohit, Nima Khalili-Tanha, Alireza Asadnia, Hamid Naderi, Ghazaleh Pourali, Zahra Yousefli, Ghazaleh Khalili-Tanha, Majid Khazaei, Mina Maftooh, Mohammadreza Nassiri, Seyed Mahdi Hassanian, Majid Ghayour-Mobarhan, Gordon A. Ferns, Soodabeh Shahidsales, Alfred King-yin Lam, Elisa Giovannetti, Elham Nazari, Jyotsna Batra, Amir Avan

**Affiliations:** 1 Metabolic Syndrome Research Center, Mashhad University of Medical Sciences, Mashhad, Iran; 2 Basic Sciences Research Institute, Mashhad University of Medical Sciences, Mashhad, Iran; 3 Medical Genetics Research Center, Mashhad University of Medical Sciences, Mashhad, Iran; 4 Department of Anesthesia, Bushehr University of Medical Sciences, Bushehr, Iran; 5 Recombinant Proteins Research Group, The Research Institute of Biotechnology, Ferdowsi University of Mashhad, Mashhad, Iran; 6 Brighton and Sussex Medical School, Division of Medical Education, Falmer, Brighton, BN1 9PH Sussex UK; 7 Cancer Research Center, Mashhad University of Medical Sciences, Mashhad, Iran; 8 Pathology, School of Medicine and Dentistry, Griffith University, Gold Coast Campus, Gold Coast, QLD 4222 Australia; 9 Department of Medical Oncology, Cancer Center Amsterdam, Amsterdam U.M.C., VU. University Medical Center (VUMC), Amsterdam, The Netherlands; 10 Cancer Pharmacology Lab, AIRC Start up Unit, Fondazione Pisana Per La Scienza, Pisa, Italy; 11 Department of Health Information, Technology and Management, School of Allied Medical Sciences, Shahid BeheshtiUniversity of Medical Science, Tehran, Iran; 12 College of Medicine, University of Warith Al-Anbiyaa, Karbala, Iraq; 13 Faculty of Health, School of Biomedical Sciences, Queensland University of Technology (QUT), Brisbane, 4000 Australia; 14 Translational Research Institute, Queensland University of Technology, Brisbane, 4102 Australia

**Keywords:** Cancer, Computational biology and bioinformatics

## Abstract

Pancreatic ductal adenocarcinoma (PDAC) is associated with a very poor prognosis. Therefore, there has been a focus on identifying new biomarkers for its early diagnosis and the prediction of patient survival. Genome-wide RNA and microRNA sequencing, bioinformatics and Machine Learning approaches to identify differentially expressed genes (DEGs), followed by validation in an additional cohort of PDAC patients has been undertaken. To identify DEGs, genome RNA sequencing and clinical data from pancreatic cancer patients were extracted from The Cancer Genome Atlas Database (TCGA). We used Kaplan–Meier analysis of survival curves was used to assess prognostic biomarkers. Ensemble learning, Random Forest (RF), Max Voting, Adaboost, Gradient boosting machines (GBM), and Extreme Gradient Boosting (XGB) techniques were used, and Gradient boosting machines (GBM) were selected with 100% accuracy for analysis. Moreover, protein–protein interaction (PPI), molecular pathways, concomitant expression of DEGs, and correlations between DEGs and clinical data were analyzed. We have evaluated candidate genes, miRNAs, and a combination of these obtained from machine learning algorithms and survival analysis. The results of Machine learning identified 23 genes with negative regulation, five genes with positive regulation, seven microRNAs with negative regulation, and 20 microRNAs with positive regulation in PDAC. Key genes *BMF*, *FRMD4A*, *ADAP2*, *PPP1R17*, and *CACNG3* had the highest coefficient in the advanced stages of the disease. In addition, the survival analysis showed decreased expression of *hsa.miR.642a*, *hsa.mir.363*, *CD22*, *BTNL9*, and *CTSW* and overexpression of *hsa.miR.153.1*, *hsa.miR.539*, *hsa.miR.412* reduced survival rate. *CTSW* was identified as a novel genetic marker and this was validated using RT-PCR. Machine learning algorithms may be used to Identify key dysregulated genes/miRNAs involved in the disease pathogenesis can be used to detect patients in earlier stages. Our data also demonstrated the prognostic and diagnostic value of *CTSW* in PDAC.

## Introduction

With 496,000 newly diagnosed cases globally and 466,000 related deaths in 2020^1^, pancreatic cancer is categorized among the malignancies with the poorest prognostic outcome^2^. According to the cancer statistics of the International Agency for Research on Cancer (IARC) GLOBOCAN, the incidence rate of pancreatic cancer has been rising in recent decades, and it accounts for 4.9% and 4.5% of worldwide cancer incidence and related deaths, respectively^1^. Pancreatic ductal adenocarcinoma (PDAC), the most common subtype of pancreatic cancer, accounts for over 90% of the cases^3^. Despite being the 10th most prevalent cancer, PDAC is the seventh most common cause of cancer-related deaths worldwide due to its poor prognosis^4^. Although the 5-year survival rate of pancreatic cancer differs regionally, it is < 10% due to a lack of clear clinical manifestations until advanced stages^5^. The primary reasons for the low survival rate of pancreatic patients are that the disease remains asymptomatic until advanced stages due to the anatomical position of the pancreas in the retroperitoneum and the lack of valuable biomarkers for early stages can be considered as other reasons^6,7^. Clinical biomarkers play a pivotal role in diagnosing and managing various cancers, including pancreatic cancer. CA-19-9 is one such biomarker commonly used for pancreatic cancer. CA-19-9 is a carbohydrate antigen that can be detected in the blood of some pancreatic cancer patients. Elevated levels of CA-19-9 may indicate the presence of pancreatic cancer, but it is important to note that this biomarker is not specific to pancreatic cancer. Other conditions, such as liver disease, bile duct obstruction, and certain gastrointestinal tumors, can also cause increased CA-19-9 levels^8^. Although the primary aetiology of pancreatic cancer has not been identified, some genes have been previously shown to be associated with the various cancer subtypes, treatment responses, or poor prognosis in pancreatic cancer^9–11^. Many cancers cannot be effectively treated in the advanced stages of disease, therefore developing novel biomarkers for the early stage is a potential approach for diagnosis, prognosis, and treatment of pancreatic cancer^12^. Currently, the *K-RAS* gene is known to be one essential gene playing a crucial role in pancreatic cancer, with a prevalence of more than 85%. Furthermore, *P53* and *P16*, as tumor suppressor genes, are inactivated in approximately 95% of pancreatic cancer patients^6^. To activate or inactivate proto-oncogenes and other related genes like those functioning as tumor suppressors, including *HER2*, *MYB*, *AKT2*, *BRCA2*, *FHIT*, *CDKN2A*, *PALB2*, *STK11*, and *PRSS1* are involved. Furthermore, the analysis of mutations in *BRCA1/2*, *MMR*(mismatch repairing system), and *NTRK1–3* fusions was performed for pancreatic cancer patients receiving the treatment of pembrolizumab, entrectinib, and larotrectinib^13^. Advanced technologies such as bioinformatics and artificial intelligence are developed to provide cancer research opportunities^14,15^. Machine learning is a part of artificial intelligence that can improve the accuracy of cancer diagnosis, prediction, and prognosis by employing various statistical techniques^16–20^.

MiRNAs are non-coding RNAs with a length of 19–24 nucleotides that regulate gene expression of more than 30% of human genes following transcription. They pair to their target's untranslated 3′(3′-UTR) region of mRNAs, resulting in inhibition or degradation of the mRNAs^21^. Up or down-regulation or misplacement of miRNAs may play crucial roles in cancer development, tumor cell proliferation, migration, invasion, and chemical resistance^22–26^. These modifications and abnormalities in the miRNA transcription levels have previously been reported in several human malignancies^27–29^. It is hypothesized that genes and miRNAs might be evaluated as biomarkers to initiate better diagnostic or predictive approaches for pancreatic cancer. Previous studies have targeted *KRAS* and other genes in pancreatic cancer. Some miRNAs, including *hsa-miR-217*, *hsa-miR-96*, *miR-216a*, and *miR-148a/b*, are reported downregulated, and some, such as the *miR-221*, *miR-210*, *miR-155*, and *miR-21* upregulated in pancreatic cancer^30–34^.

**Figure 1:**
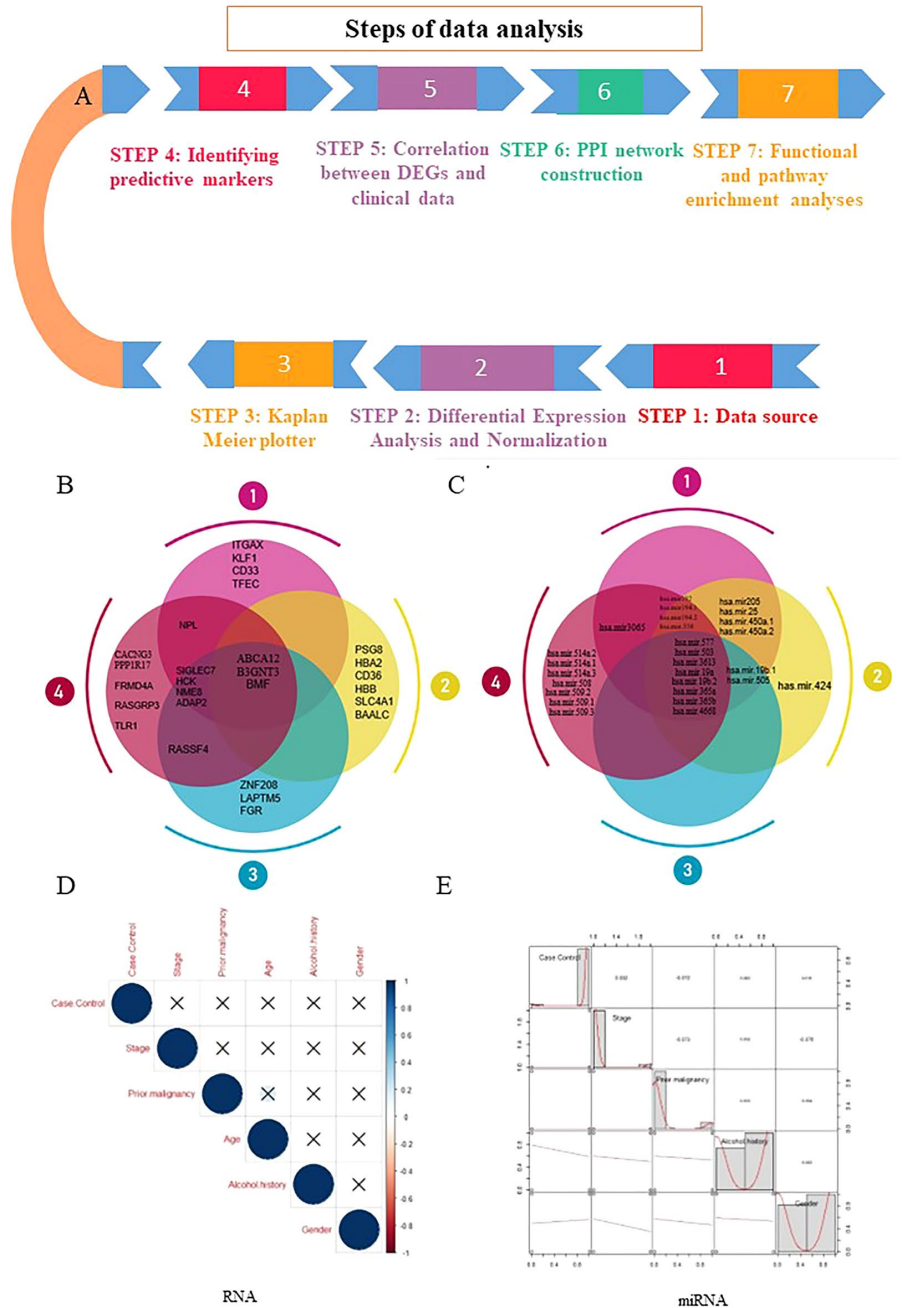
(**A**) The overall workflow, (**B**, **C**) miRNAs and important genes identified by machine learning in PDAC at different stages. (**D**, **E**) The association of clinical variables with cancer that were not significant.

The Cancer Genome Atlas (TCGA) is a project that maps out the genome variation of human cancerous cells by RNA sequencing and using a non-malignant cell as a reference. These maps have identified many core genetic pathways activated in various cancers^35,36^. Therefore, in the current study, we performed gene expression proofing of pancreatic cancer using the TCGA database and Machine learning to identify differential expression genes (DEGs) and differentially expressed miRNAs (DEmiRNA). Survival was assessed using Kaplan–Meier analysis to predict prognostic biomarkers and the risk model. Additionally, the protein–protein interaction (PPI), the molecular pathways, the co-expression of DEGs, and the correlation between candidate genes and pancreatic cancer with clinical data were evaluated. Furthermore, the diagnostic markers were detected based on machine learning technology (Fig. 1A).

## Material and method

### Data collection

The TCGA database (http://tcga-data.nci.nih.gov/tcga/) was utilized to extract pancreatic cancer gene and miRNA data from 183 and 193 samples including healthy and tumor samples. RNA gene expression, microRNA, and clinical data were downloaded.

### Data preprocessing and the identification of DEGs (differential expression genes)

In the pre-processing step, gene expression data were filtered to eliminate the gene and miRNA with zero expression and duplicates. Then, data was normalized with Limma and DESEQ2 packages in R 4.0.3 software. Filtering and normalization are the most important step in data analysis performed before machine learning. Genes and miRNAs were adjusted between pancreatic cancer samples and healthy tissue samples based on the particular criteria, including *P <* 0.05 and − 1.5 <|Log2FC (fold change) |< 1.5, to evaluate the upregulated and downregulated genes of the data integrity and subsequent analysis. The heatmap was created by “cluster, dendextend, circlize, RcolorBrewer, ComplexHeatmap, d3heatmap, gplots, pheatmap, and gplots” packages in R software version 4.3.1.

### Identifying predictive markers

Machine learning methods can be used to analyze the data collected from various biological data, such as genomics, transcriptomics, and metabolic data^27^. Our study used machine learning algorithms, including Random Forest (RF), Max Voting, Adaboost, Gradient Boosting machines(GBM), and Extreme Gradient Boosting (XGB), for the analysis of DEGs and identifying novel biomarkers.

#### Machine learning by stage

##### Ensemble learning

This method performs better than using a simple algorithm alone because it employs many algorithms to provide poor predictive outcomes in accordance with the features taken from various estimations of data and integrates the results using various voting methods^37^.

**Random Forest (RF):** A technique that involves a set of decision trees that naturally incorporate feature selection and interactions into the learning process and report their average as an acceptable label. This algorithm is also the most popular machine learning method.

##### Max voting

One of the well-known methods in decision-making is max voting. This process is done independently, and as the best class vote is estimated, the outcome with the highest vote is carried out^38^.

##### Adaboost

One of the most efficient recognition algorithms in machine learning is Adaptive Boosting, aka. Adaboost. This algorithm makes a pile of weak learners by keeping a set of weights over training data and modifying them after each weak cycle adaptively to make more precise and strong learners out of a collection of weak learners. This recognition algorithm is used for ensemble learning as it has outstanding classification performance that is beneficial in estimating fruit biochemical parameters, image recognition, and complex change prediction modeling^39^.

**Gradient boosting machines(GBM):** As decision trees develop, a group forms gradient boosting machines using the information previously generated by growing trees. This way, each decision tree stems from an original training set focused on the parts where earlier model iterations deliver poor prediction^40^.

**Extreme Gradient Boosting (XGB):** Extreme Gradient Boosting (XGB) is considered one of the applications of gradient boosted decision trees. To have optimized memory usage and get the most out of hardware computing power, we can use XGBoost. It also reduces the processing time with enhanced performance compared to other machine learning algorithms and deep learning models^41^.

### Performance of machine learning methods

In both true positive and true negative machine learning, accuracy is a measure of an algorithm's effectiveness and performance. F1score is a measure mostly used in unbalanced data to evaluate the algorithm's performance in a false positive and false negative. Auc_curve is a measure to evaluate the correct performance of the algorithm in classifying each class. The confusion matrix is a table that identifies four types of classifications (TN, TP, FN, FP) and shows the algorithm's overall performance. R^2^ is mainly used in regression algorithms to evaluate the performance of machine learning methods.

### Investigation of the correlations of Clinical/Demographic with cancer

To explore the relationships between variables, R 4.1.3 was used to create a cancer correlation matrix to investigate the association between clinical data, including age, tumor size, lymph node involvement, distant metastasis, and stage. A correlation matrix visualizes connections by showing the coefficient of correlation between variables. The correlation coefficient is evaluated on a scale of − 1 to 1. While a negative correlation shows the variables moving in opposite directions, a positive correlation indicates that the variables are moving in the same direction. The cut-off for statistical significance was set ata *p <* 0.05.

### Functional enrichment analysis of the genes and miRNAs

Functional analysis of Gene Ontology (GO) and Reactom, Do, GSEA pathways signaling pathways was performed. In these two analyzes, the categories that include biological process (BP), cellular components (CC), and molecular function (MF) are used. Results with enrichment score > 1, FDR < 0.25, and adjust *p <* 0.05 were determined as statistically significant results.

### PPI network construction

The STRING v11.5 database (http://string-db.org/) was obtained to evaluate the interactions between the target genes of the selected miRNAs. The highest confidence score was set at 70.7 and was considered significant. Proteins were selected based on their interaction with other proteins. Cytoscape software was utilized to view the protein–protein interaction networks (PPIs). Selected miRNAs with several connections to other target genes propose their essential part in PPI.

### Identifying prognostic markers

Kaplan‐Meier survival curves and Cox proportional hazard ratio (HR) were plotted for top-selected genes and miRNAs using SPSS version 20 and 95% CI . All the data were analyzed under screening criteria, including the cut-off threshold of HR > 1 and *P <* 0.05. The candidate genes and miRNAs presented as “prognostic genes”.

### Identifying diagnostic markers

Diagnosing PDAC before the tumor spreads provides the best chance for treatment and survival. Here, we assessed the candidate genes, miRNAs, and every combination discovered through survival analysis and machine learning algorithms. In order to evaluate the diagnostic potency and create diagnostic models, a generalized linear model, and combined receiver operating characteristic (ROC) curve analysis were used. Additional diagnostic parameters such as sensitivity, specificity, cut-off value, positive predictive value, negative predictive value, and area under the ROC curve were assessed to evaluate the discrimination of individual or combined biomarkers. The entire procedure was applied using R 4.1.3’s combioROC package.

### Quantitative real-time PCR

RNA was isolated from twenty-one Formalin-Fixed Paraffin-Embedded (FFPE) tissue samples using a Parstous kit (Parstous, Tehran, Iran). The extraction quality was evaluated on 1.5% agarose gel, and the quantity was assessed by a Nanodrop 2000 spectrophotometer (BioTek, USA EPOCH). cDNA was synthesized according to the manufacturer's instructions (Parstous, Tehran, Iran). Quantitative real-time PCR was performed using specific primers (Macrogene Co., Seoul, South Korea) and SYBR green master mix (Parstous Co. Tehran, Iran) using an ABI-PRISM StepOneTM instrument (Foster City, CA)^18^. To identify tissue-specific housekeeping genes for gene expression analysis and to avoid single control normalization error, accurate normalization of qRT-PCR data based on the geometric means of multiple internal control genes was performed. The housekeeping gene which was used as an internal control was GAPDH.

### Statistical analysis

The RNA-Seq data analysis, including quality control, preprocessing, and identifying differential expression genes, was performed by R software version 4.3.1. The data were compared by paired t-test and were expressed as mean ± standard deviation (SD). A p-value < 0.05 was considered statistically significant.

### Ethics approval and consent to participate

The data was downloaded from TCGA portal (https://tcga-data.nci.nih.gov/). TCGA generates over 2.5 petabytes of genomic, epigenomic, transcriptomic, and proteomic data. The data will remain publicly available for anyone in the research community to all procedures consisting of Ethical issues followed by the TCGA committee. This article does not contain any studies with animals performed by any of the authors. This study was approved by the Ethical Committee of Mashhad University of Medical Sciences (IR.MUMS.MEDICAL.REC.1401.430).

## Results

### Data description and Identification of differentially expressed genes (DEGs) and differentially expressed miRNAs (DEmiRNA)

The clinical features of the patients are shown in Supplement Table 1. TCGA database containing 193 patients was used as a source to download our required data. Then the data were filtered and finally normalized with the DEseq package. Genes compliant with criteria 1 | LogFC |> and *p*-value < 0.05 were selected. Using five different machine learning methods, including SVM, DTS, RF, LR, and KNN, some key genes were nominated and analyzed by five various criteria: Accuracy, f1score, r2score, auc_curve, and Confusion matrix. During each step, the best classification algorithm was introduced.

### Identifying predictive markers for genes and miRNAs

As shown in Fig. 1B,C, three genes (*ABCA12*, *B3GNT3*, and *BMF*) and eight miRNA (*hsa.miR.577*, *hsa.miR.503*, *hsa.miR.3613*, *hsa.miR.19a*, *hsa.miR.19b.2*, *hsa.miR.365a*, *hsa.miR.365b*, and *hsa.miR.4668*) were found to be dysregulated in four different stages of pancreatic cancer.

### Investigation of the correlations of Clinical/Demographic with cancer

**Figure 2:**
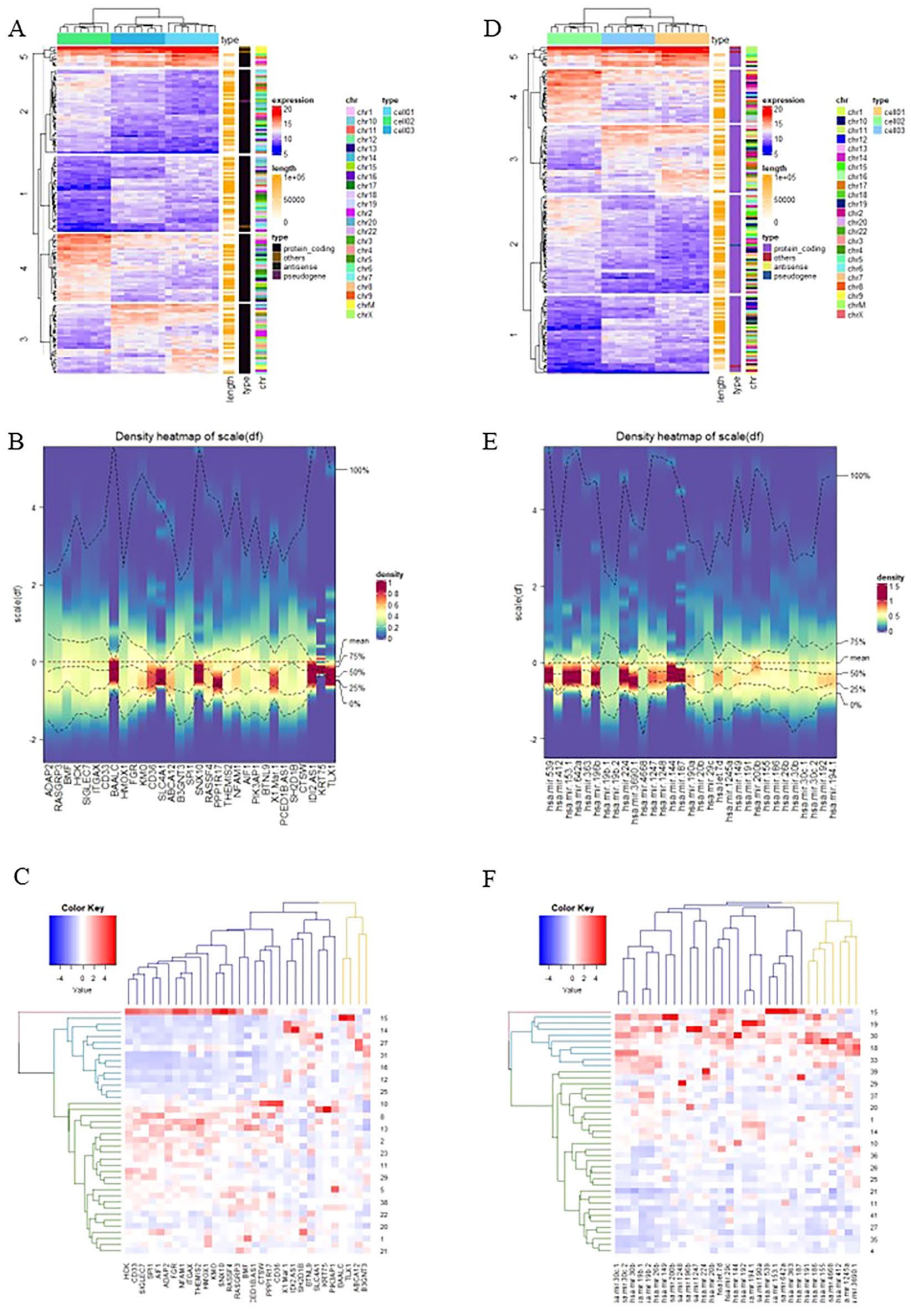
(**A**–**C**) The heatmaps of dysregulated genes, (**D**–**F**) The heatmaps of dysregulated miRNAs (created by “cluster, dendextend, circlize, RcolorBrewer, ComplexHeatmap, d3heatmap, gplots, pheatmap, and gplots” packages in R software version 4.3.1.).

No association was found between DEG and clinical data for the patients from whom the RNA samples were obtained; only age was significantly associated with prior malignancy. The correlation is considered low when less than 0.3, moderate between 0.3 and 0.6, and strong when more than 0.6 (Fig. 1D,E). The heat map depicted for visualizing DEGs and DEmiRNA across the samples based on the specific criteria (Fig. 2A–F).

### Functional enrichment analysis of the RNAs and miRNAs

**Figure 3:**
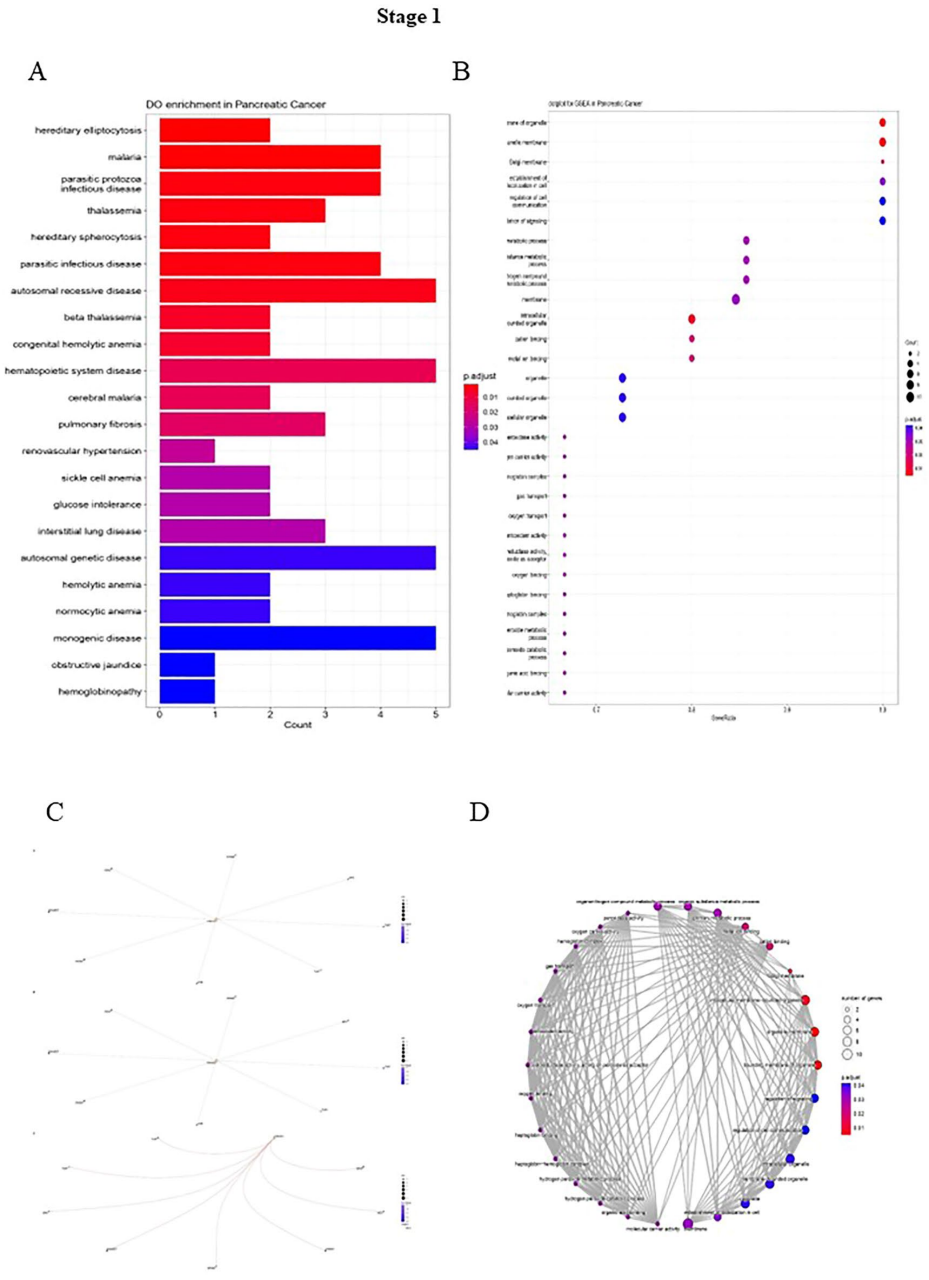
(**A**) DO functional annotation in stage 1 of PDAC. (**B**) GSEA functional annotation in stage 1 of PDAC. (**C**) GO functional annotation in stage 1 of PDAC. (**D**) Reactome functional pathways in stage 1 of PDAC. The P-value is less than 0.05 and is shown by the color.

**Figure 4:**
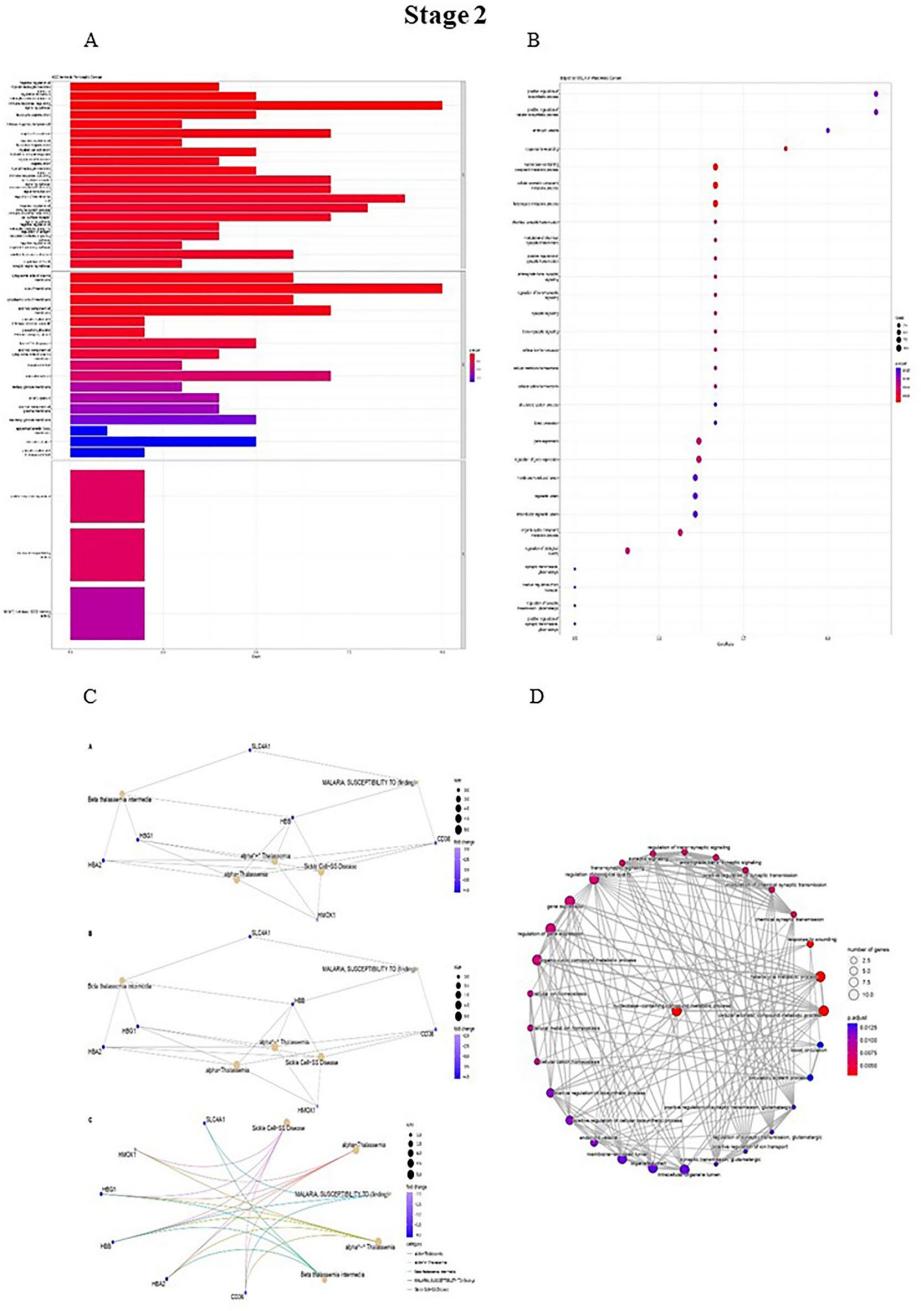
(**A**) DO functional annotation in stage 2 of PDAC. (**B**) GSEA functional annotation in stage 2 of PDAC. (**C**) GO functional annotation in stage 2 of PDAC. (**D**) Reactome functional pathways in stage 2 of PDAC. The P-value is less than 0.05 and is shown by the color.

**Figure 5:**
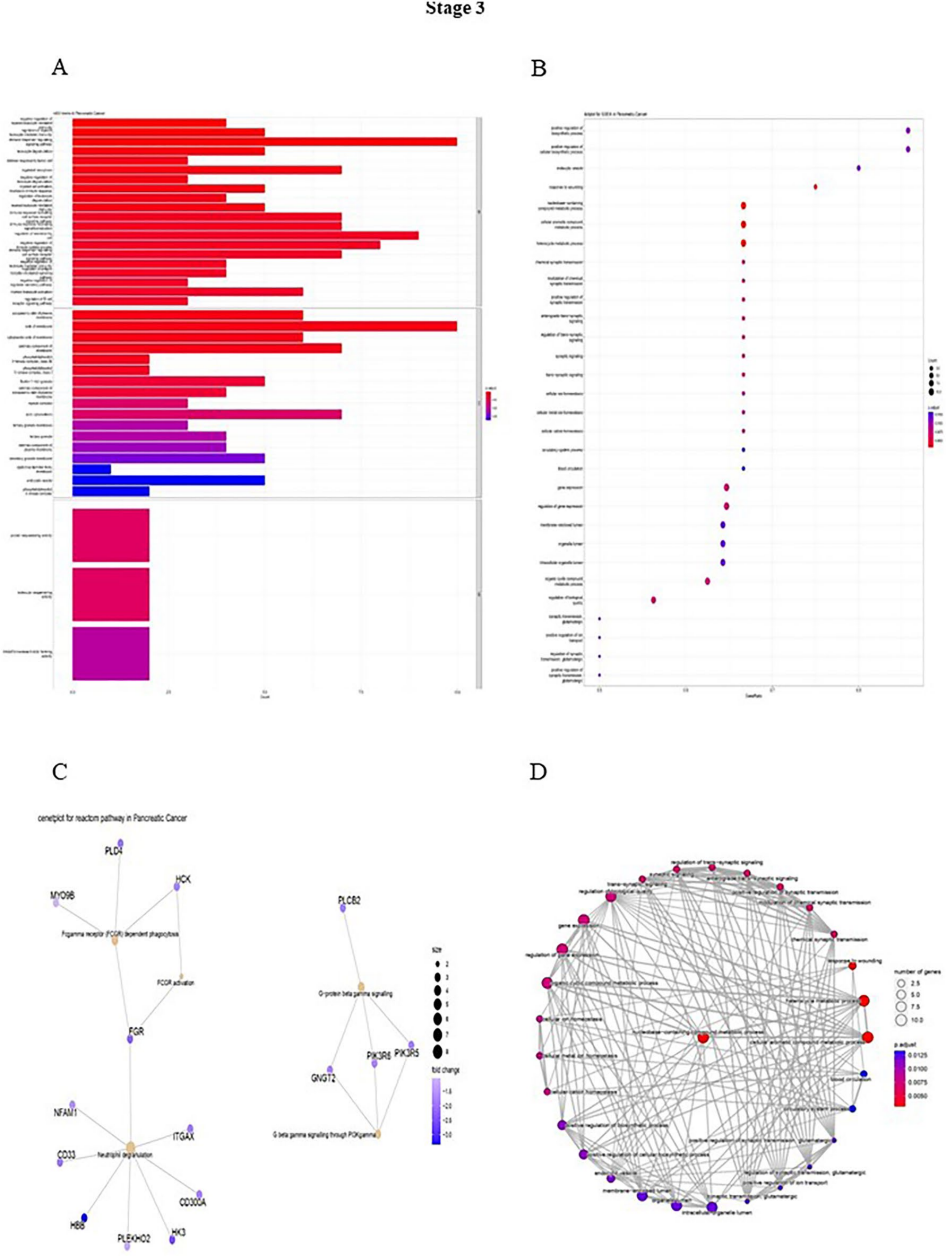
(**A**) DO functional annotation in stage 3 of PDAC. (**B**) GSEA functional annotation in stage 3 of PDAC. (**C**) GO functional annotation in stage 3 of PDAC. (**D**) Reactome functional pathways in stage 3 of PDAC. The P-value is less than 0.05 and is shown by the color.

**Figure 6:**
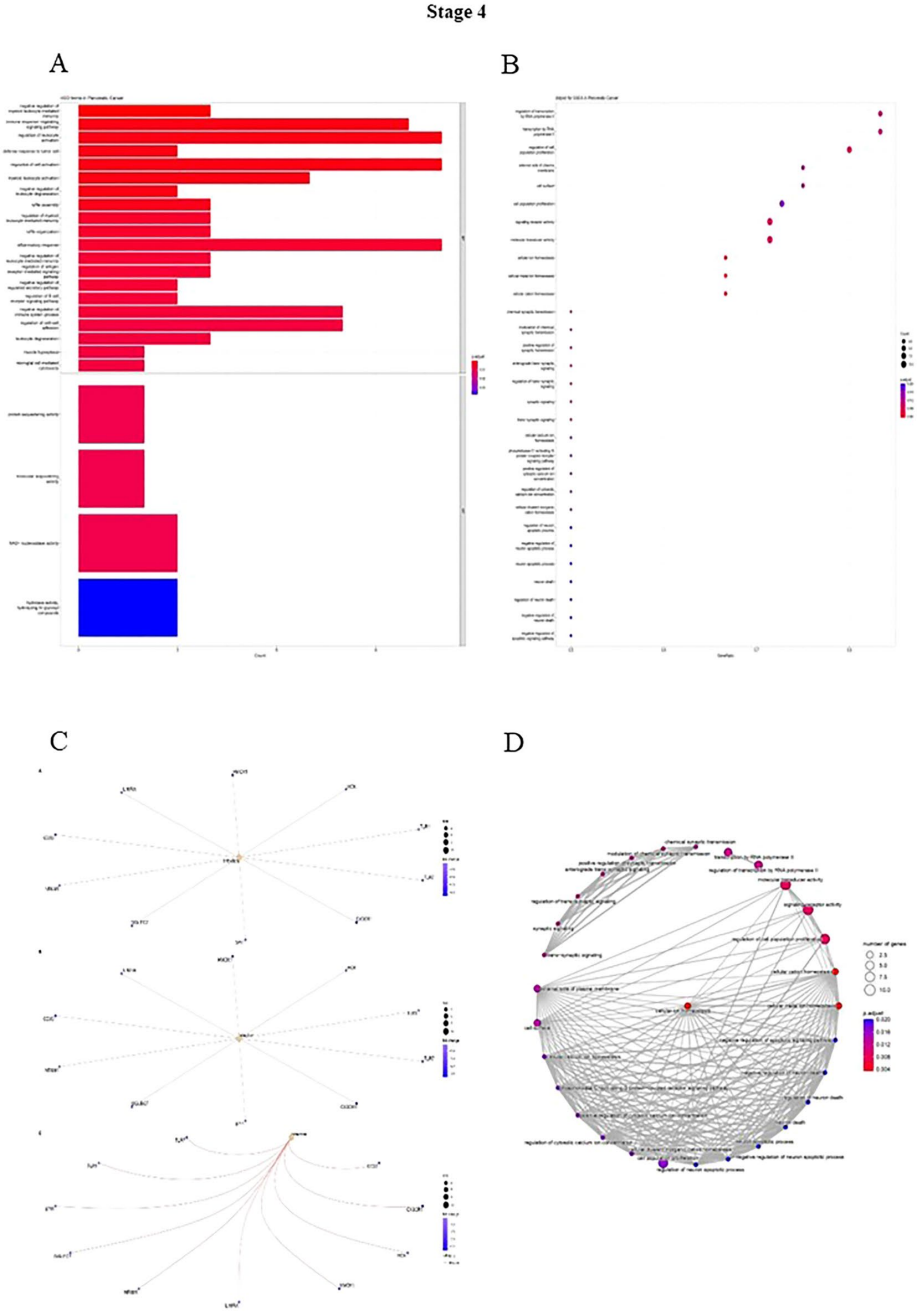
(**A**) DO functional annotation in stage 4 of PDAC. (**B**) GSEA functional annotation in stage 4 of PDAC. (**C**) GO functional annotation in stage 4 of PDAC. (**D**) Reactome functional pathways in stage 4 of PDAC. The P-value is less than 0.05 and is shown by the color.

A list of genes was generated, then the gene enrichment to determine the functionally related genes involved in different pathways was calculated, and the expression of other genes was adjusted by R software. Finally, the key genes were enriched to study the Reactom, Do, Go, GSEA pathways. In stage 1, the highest number of genes in the biological process (BP) portion is involved in regulating leukocyte activation and cell activation. As in cellular component (CC), most genes play a role in the receptor complex and side of the membrane pathways. Moreover, during stage 1 in the Molecular Function (MF), the highest number of genes are involved in the pathways of NAD + nucleosidase activity and hydrolyzing N-glycosyl compounds hydrolase activity (Fig. 3). In stage 2, most genes in the BP were involved in the positive regulation of cell death. In CC, the two pathways of hydrolyzing N-glycosyl mitochondrial membrane and mitochondrial envelope are affected by most of the genes, and in MF, the highest number of genes are involved in oxidoreductase activity (Fig. 4). During stage 3, in the BP portion, the highest number of genes are in the immune response-regulating signaling pathway; in the CC stage, the highest number of genes in the side of the membrane pathway, and in the MF section, the genes are equally involved in 3 molecular pathways including, protein and molecular sequestering activity, and NAD(P) H oxidase H_2_O_2_-forming activity (Fig. 5). Stage 4 in the BP involved three inflammatory response pathways, cell activation and activation regulation of leukocytes. Also, in the CC, the highest number of genes are involved in the two pathways of hydrolyzing N-glycosyl compounds, hydrolase activity, and NAD + nucleosidase activity (Fig. 6).

### PPI network construction

**Figure 7:**
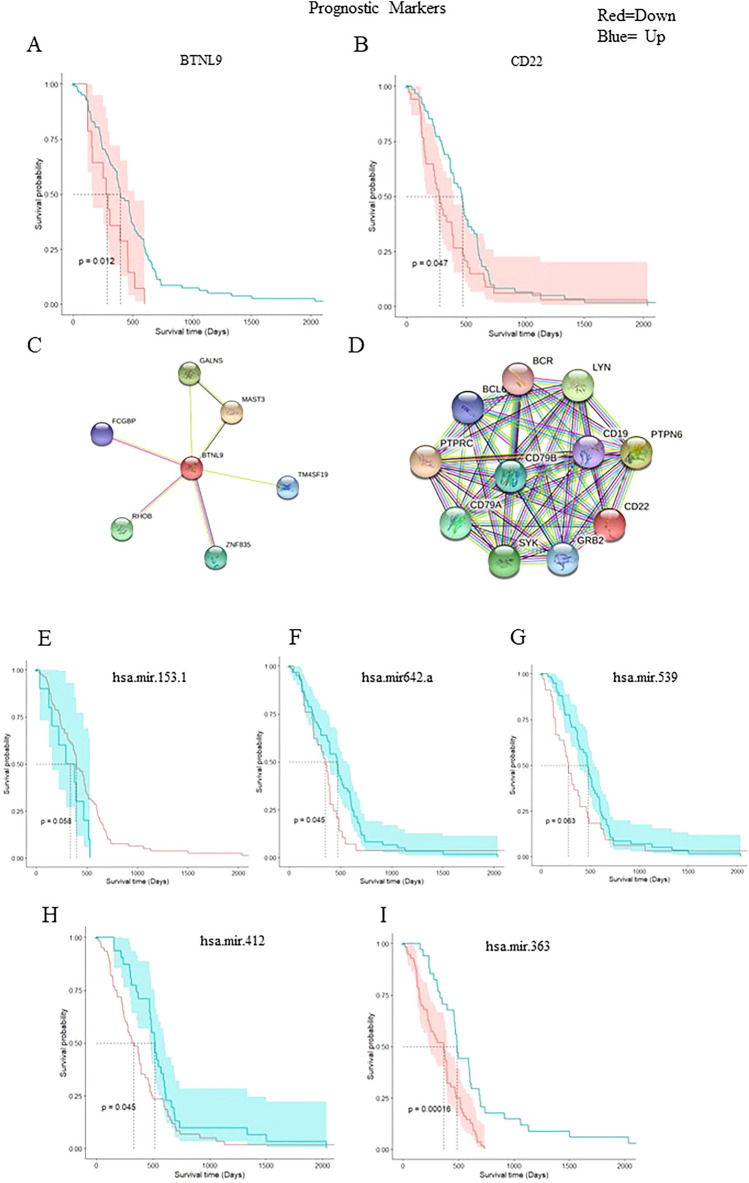
(**A**, **B**, **E**, **F**, **G**, **H**, **I**) Kaplan–Meier plots of prognostic genes and miRNAs, (**C**, **D**) PPI network of novel genes from STRING.

**Figure 8:**
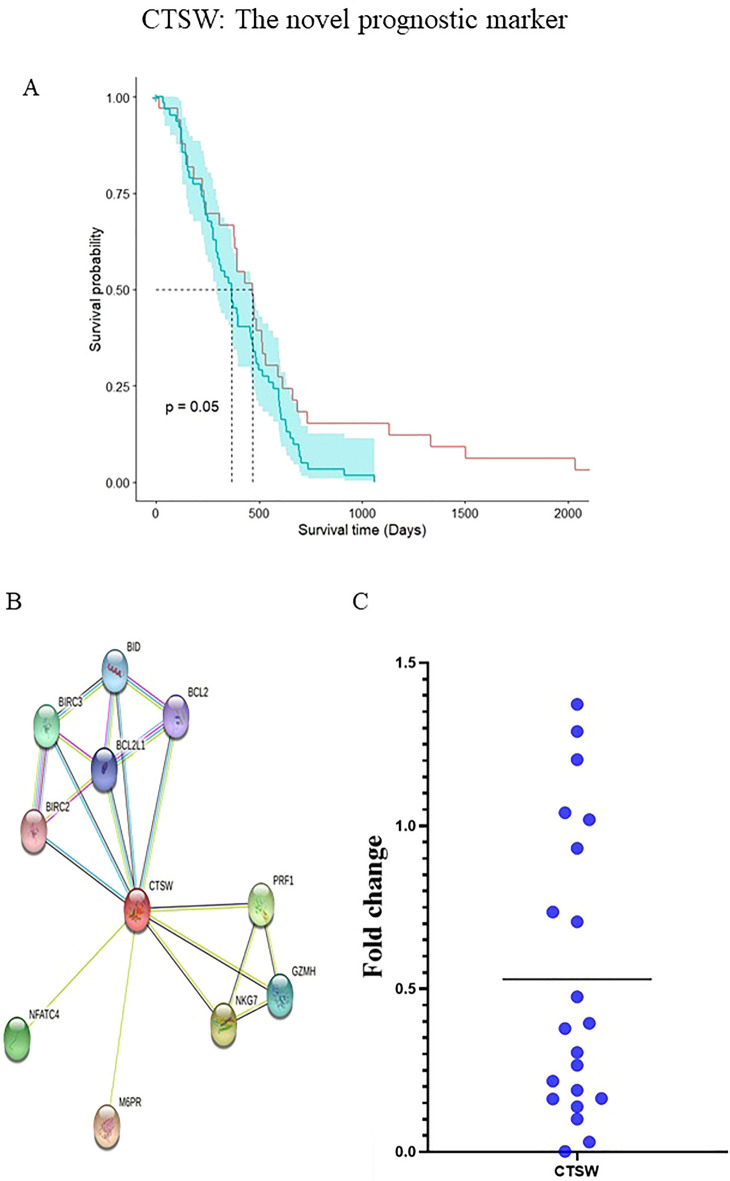
(**A**) Kaplan–Meier plot of *CTSW* gene, (**B**) PPI network of *CTSW* gene from STRING, (**C**) The level of *CTSW* in PDAC tumor tissue, as detected by RT-PCR.

Figure 7 illustrates the interaction of DEGs checked and plotted using the STRING (interaction score: 0.4). In accordance with PPI network, the *CD22* gene has the highest binding capacity, followed by *CTSW* and *BTNL9* (Figs. 7C,D, 8B).

### Identifying prognostic markers for RNAs and miRNAs

Kaplan Meier analysis was applied to identify key prognostic signature genes in pancreatic cancer. The outcome revealed survival is associated with three genes, including *BTNL9* (HR = 1.02), *CD22* (HR = 1.7), and *CTSW* (HR = 2.03) and five miRNAs, including *hsa.miR.539* (HR = 1.3), *hsa.miR.412* (HR = 1.04), *hsa.miR.153.1* (HR = 1.5), *hsa.miR.642a* (HR = 1.00), and *hsa.miR.363* (HR = 1.5) in PDAC patients. All analyses were performed by R software (Figs. 7, 8A).

### Identifying diagnostic markers for RNAs and miRNAs

For stages 1 and 2, GLM model analysis for HCK and SIGLEC7 combination in diagnostic biomarkers with coefficients of 1.2920 and − 0.5562 (AUC of 0.74, 95% CI with sensitivity of 0.85 and specificity of 0.66). For stage 3, the combination of *B3GNT3*, *ABCA12*, and *ADAP2* with 0.8409 (AUC of 0.86, 95% CI with sensitivity of 0.8 and specificity of 1. In stage 4, our finding showed that the Coefficients of combination AIF1 and RASGRP3 were 4.233 and − 7.841 (AUC of 0.86, 95%CI with 0.8 sensitivity and one specificity). Furthermore, three miRNAs (Has.mir.194.2, hsa.mir.194.1, and hsa.mir.192) had the highest AUC value, sensitivity, and specificity and coefficients of 4.932, 5.531, and 3.584, respectively (Supplement Table 1).

### Validation of CTSW in an additional cohort of PDAC

The clinical data are shown in Supplement Table 2; our population consisted of 52.4% males and 47.6% females. The mean age was 61.66 years and 52.4% underwent advanced stage. We further evaluated the value expression of *CTSW* in PDAC cases using RT-PCR. This data showed the significant downregulation of this gene in tumor tissue (*P <* 0.05) (Fig. 8C).

## Discussion

To the best of our knowledge, this is the first study showing the potential of downregulation of *hsa.miR.642a*, *hsa.mir.363*, *CD22*, *BT*NL9, and *CTSW* and overexpression of *hsa.miR.153.1*, *hsa.miR.539*, *hsa.miR.412* with shorter survival of patients with PDAC (Supplement Fig. 1) The result indicated the diagnostic value of the combination of *AIF1* and *RASGRP3* in an advanced stage with the Coefficients of combination *AIF1* and *RASGRP3* were 4.233 and -7.841 (AUC of 0.86, 95%CI with 0.8 sensitivity and one specificity). The result of the survival analysis showed that the *CTSW* gene is a novel prognostic marker. *CTSW* (Cathepsin W), also known as *LYPN* is a novel human cysteine proteinase member of the C1 peptidase family expressed in CD8 + T and NK cells and regulated by interleukin-2. This gene has a specific function in the cytotoxicity-mediated mechanism by NK cells and CD8 + T cells. Various T cell populations can act differently in regulating a tumor's degree, stage, and ability to invade endometrial cancer. *CTSW* is an immunomodulatory gene that functions similarly to the *CTSF* gene^42^. In research done by Song and colleagues, the expression of *CTSF* in non-small cell lung cancer was evaluated, and downregulated levels of *CTST* were observed in NSCLC samples despite normal tissues and good prognosis of NSCLC being correlated with high expression of *CTSF*. Besides using GeneMANIA, the gene–gene interaction network was established for *CTSF* and showed that *CTSF* had a similar function as *CTSW* genes^43^. A study on endometrial cancer reported that the *CTSW* gene had a positive correlation with tumor infiltration levels of B cells, CD8 + T cells, CD4 + T cells, macrophages, and dendritic cells^42^.

*BMF* (Bcl-2 modifying factor) is a proapoptotic protein that belongs to the BCL-2 protein family. This gene has been identified in the BH3-only proteins subgroup and initiates the innate apoptotic pathway^44^. Consequently, *BMF* is linked with various cellular activities, including chemical sensitivity. For example, the *YAP*/*TEAD*/*SLUG* axis suppressed apoptosis by suppressing *BMF* transcription^45^. Badr et al. reported that upregulation of livin and downregulation of *BMF* and *p53* expression are significantly correlated with more tumor aggressiveness (advanced TNM stage), making metastasis progress more rapidly and decreasing overall survival in colon cancer patients. Thus, we can apply these genes as crucial prognostic markers related to poor results^46^. Another research showed that STARD13 3′UTR could play as a ceRNA for *BMF* to enhance apoptosis and be used as a potential therapeutic target in breast cancer cells^47^. FERM is a superfamily of proteins, and one of its members is FERM domain-containing 4A (FRMD4A); these proteins are ubiquitous parts of the cytocortex and are involved in cell transport cell structure and signaling functions. Moreover, tumor progression and metastasis are the cellular events in which the proteins of the FERM family are involved. These proteins function as regulators or scaffolding units and are involved in many membrane-associated factors' functions^48^. In another study on tongue squamous cell carcinoma and squamous cell carcinoma, the expression of *FMRD4A* was increased, contrasting our findings.^48,49^. ArfGAP with dual PH domains 2 (ADAP2) belongs to the *ArfGAP* family of genes, which is the GTPase activating protein. This gene is expressed for ARF6, which acts as a scaffold in the innate and membrane immunosuppressive phosphate signaling pathways^50^. It is reported that the *ADAP2* gene expression was decreased in primary lower-grade glioma^51^. Contrary to this, the expression of this gene was increased in radiation-resistant esophageal cancer cells^52^. Protein phosphatase 1 regulatory subunit 17(PPP1R17), also known as C7orf16, is a negative regulator that inhibits phosphatase activities of protein phosphatase 1 (PP1) and protein phosphatase 2A (PP2A) complexes which their substrates are the S6 ribosomal protein^53^. Contrary to our results, research in lung cancer adenocarcinoma has demonstrated that PPP1R17 can be used as biomarkers as it was specifically detected in stage III, which can help us detect cancer stage in tumor progression through cleft junction incompatibility, Wnt signaling, and GPCR signaling pathways^54^. Another study reported that PPP1R17 is a HAR-regulated gene that slows the progression of the neural precursor cell cycle while increasing cell cycle length, which is mainly observed in the neural growth of primates, especially humans.^55^. The *CACNG3* gene encodes a transient AMPA regulatory protein (TARP) known as an auxiliary subunit of the calcium channel γ3. This gene is involved in the neurons formation, and has also been reported as a potential source of epilepsy^56^. In line with our results, several other studies have shown that the *CACNG 3* gene in Gliomas has been predicted as an oncogene and significantly dysregulated in glioblastoma tissue compared to healthy controls^57^. Other studies have also reported dysregulation of CACNG3 gene in breast cancer^58^. In our study, *hsa.miR.153.1*, also known as *MIRN153-1*, was found to be a new microRNA that had not been used in any other diseases or cancers and had increased expression in pancreatic cancer.

In conclusion, we have identified some specific genes that are differentially expressed at different stages of pancreatic cancer. *CTSW* gene was reported as a novel prognostic biomarker and validated by Real-time PCR in pancreatic tumor tissue. Eventually, we highly recommend using machine learning to detect biomarkers in other cancers as well.

### Supplementary Information

Supplementary Information.

## Data Availability

The data was downloaded from TCGA portal (https://tcga-data.nci.nih.gov/). TCGA generated over 2.5 petabytes of genomic, epigenomic, transcriptomic, and proteomic data. The data will remain publicly available for anyone in the research community.

## References

[1] Ferlay J, Colombet M, Soerjomataram I, Parkin DM, Piñeros M, Znaor A (2021). Cancer statistics for the year 2020: An overview. Int. J. Cancer.

[2] Jagadeesan B, Haran PH, Praveen D, Chowdary PR, Aanandhi MV (2021). A comprehensive review on pancreatic cancer. Res. J. Pharm. Technol..

[3] Jin C, Bai L (2020). Pancreatic cancer—Current situation and challenges. Gastroenterol. Hepatol. Lett..

[4] Menini S, Iacobini C, Vitale M, Pesce C, Pugliese G (2021). Diabetes and pancreatic cancer—A dangerous liaison relying on carbonyl stress. Cancers.

[5] Hu JX, Zhao CF, Chen WB, Liu QC, Li QW, Lin YY (2021). Pancreatic cancer: A review of epidemiology, trend, and risk factors. World J. Gastroenterol..

[6] Kamisawa, T., Wood, L.D., Itoi, T., & Takaori, K.J.T.L. Pancreatic Cancer. *Lancet*. **388**(10039), 73–85 (2016). 10.1016/S0140-6736(16)00141-0 26830752

[7] Kanno A (2018). Multicenter study of early pancreatic cancer in Japan. Pancreatology.

[8] Ballehaninna UK, Chamberlain RS (2013). Biomarkers for pancreatic cancer: Promising new markers and options beyond CA 19-9. Tumor Biol..

[9] Jones S, Zhang X, Parsons DW, Lin JC-H, Leary RJ, Angenendt P (2008). Core signaling pathways in human pancreatic cancers revealed by global genomic analyses. Science.

[10] Yang J, Shi W, Zhu S, Yang C (2020). Construction of a 6-gene prognostic signature to assess prognosis of patients with pancreatic cancer. Medicine.

[11] Waddell N, Pajic M, Patch A-M, Chang DK, Kassahn KS, Bailey P (2015). Whole genomes redefine the mutational landscape of pancreatic cancer. Nature.

[12] De Dosso S, Siebenhüner AR, Winder T, Meisel A, Fritsch R, Astaras C (2021). Treatment landscape of metastatic pancreatic cancer. Cancer Treat. Rev..

[13] Nevala-Plagemann C, Hidalgo M, Garrido-Laguna I (2020). From state-of-the-art treatments to novel therapies for advanced-stage pancreatic cancer. Nature Rev. Clin. Oncol..

[14] Kolodziejczyk AA, Kim JK, Svensson V, Marioni JC, Teichmann SA (2015). The technology and biology of single-cell RNA sequencing. Mol. Cell.

[15] Chinnappan J (2021). Integrative bioinformatics approaches to therapeutic gene target selection in various cancers for nitroglycerin. Sci. Rep..

[16] Hornbrook MC, Goshen R, Choman E, O’Keeffe-Rosetti M, Kinar Y, Liles EG (2017). Early colorectal cancer detected by machine learning model using gender, age, and complete blood count data. Dig. Dis. Sci..

[17] Kinar Y, Akiva P, Choman E, Kariv R, Shalev V, Levin B (2017). Performance analysis of a machine learning flagging system used to identify a group of individuals at a high risk for colorectal cancer. PLoS ONE.

[18] Dimitriou N, Arandjelović O, Harrison DJ, Caie PD (2018). A principled machine learning framework improves accuracy of stage II colorectal cancer prognosis. NPJ Digit. Med..

[19] Nazari E, Pourali G, Khazaei M, Asadnia A, Dashtiahangar M, Mohit R (2023). Identification of potential biomarkers in stomach adenocarcinoma using machine learning approaches. Curr. Bioinform..

[20] Khalili-Tanha, G. *et al*. Identification of ZMYND19 as a novel biomarker of colorectal cancer: RNA-sequencing and machine learning analysis. *J. Cell Commun. Signal.* 1–17. 10.1007/s12079-023-00779-2 (2023). 10.1007/s12079-023-00779-2 PMC10713961 37428302

[21] Salmaninejad A, Pourali G, Shahini A, Darabi H, Azhdari S (2022). MicroRNA and exosome in retinal-related diseases: Their roles in the pathogenesis and diagnosis. Comb. Chem. High Throughput Screen..

[22] Yonemori K, Kurahara H, Maemura K, Natsugoe S (2017). MicroRNA in pancreatic cancer. J. Hum. Genet..

[23] Waspada I, Wibowo A, Meraz NS (2017). Supervised machine learning model for microrna expression data in cancer. Jurnal Ilmu Komputer dan Informasi.

[24] Savareh BA, Aghdaie HA, Behmanesh A, Bashiri A, Sadeghi A, Zali M (2020). A machine learning approach identified a diagnostic model for pancreatic cancer through using circulating microRNA signatures. Pancreatology.

[25] Shi X-H, Li X, Zhang H, He R-Z, Zhao Y, Zhou M (2018). A five-microRNA signature for survival prognosis in pancreatic adenocarcinoma based on TCGA data. Sci. Rep..

[26] Samami E, Pourali G, Arabpour M, Fanipakdel A, Shahidsales S, Javadinia SA (2022). The potential diagnostic and prognostic value of circulating MicroRNAs in the assessment of patients with prostate cancer: rational and progress. Front. Oncol..

[27] Xia T, Chen X-Y, Zhang Y-N (2021). MicroRNAs as biomarkers and perspectives in the therapy of pancreatic cancer. Mol. Cell. Biochem..

[28] Acunzo M, Romano G, Wernicke D, Croce CM (2015). MicroRNA and cancer—A brief overview. Adv. Biol. Regulat..

[29] Pourali G, Khalili-Tanha G, Nazari E, Maftooh M, Nassiri M, Hassanian SM (2023). Circulating tumor cells and cell-free nucleic acids as biomarkers in colorectal cancer. Curr. Pharm. Des..

[30] Xue Y, Abou Tayoun AN, Abo KM, Pipas JM, Gordon SR, Gardner TB (2013). MicroRNAs as diagnostic markers for pancreatic ductal adenocarcinoma and its precursor, pancreatic intraepithelial neoplasm. Cancer Genet..

[31] Sohrabi E, Rezaie E, Heiat M, Sefidi-Heris Y (2021). An integrated data analysis of mRNA, miRNA and signaling pathways in pancreatic cancer. Biochem. Genet..

[32] Khojasteh-Leylakoohi, F. *et al*. Association of a genetic variant in the adenosine triphosphate transmembrane glycoprotein and risk of pancreatic cancer. *Ann. Pancreatic Cancer*. **6**, 6 (2023).

[33] Akhlaghipour, I., Fanoodi, A., Zangouei, A.S., Taghehchian, N., Khalili-Tanha, G. & Moghbeli, M. MicroRNAs as the critical regulators of forkhead box protein family in pancreatic, thyroid, and liver cancers. *Biochem. Genetics* **61**(5), 1645–1674 (2023). 10.1007/s10528-023-10346-4 36781813

[34] Sardarzadeh N, Khojasteh-Leylakoohi F, Damavandi S, Khalili-Tanha G, Dashtiahangar M, Khalili-Tanha N (2022). Association of a genetic variant in the cyclin-dependent kinase inhibitor 2B with risk of pancreatic cancer. Rep. Biochem. Mol. Biol..

[35] Tomczak K, Czerwińska P, Wiznerowicz M (2015). The cancer genome atlas (TCGA): An immeasurable source of knowledge. Contemp. Oncol..

[36] Azari H, Nazari E, Mohit R, Asadnia A, Maftooh M, Nassiri M (2023). Machine learning algorithms reveal potential miRNAs biomarkers in gastric cancer. Sci. Rep..

[37] Dong X, Yu Z, Cao W, Shi Y, Ma Q (2020). A survey on ensemble learning. Front. Comp. Sci..

[38] Usman, M., Shafique, Z., Ayub, S. & Malik, K. Urdu text classification using majority voting. *Int. J. Adv. Comput. Sci. Appl*. **7**(8). 10.14569/IJACSA.2016.070836 (2016).

[39] Wang J, Xue W, Shi X, Xu Y, Dong C (2021). Adaboost-based machine learning improved the modeling robust and estimation accuracy of pear leaf nitrogen concentration by in-field VIS-NIR spectroscopy. Sensors.

[40] Baran Á, Lerch S, El Ayari M, Baran S (2021). Machine learning for total cloud cover prediction. Neural Comput. Appl..

[41] Dhieb, N., Ghazzai, H., Besbes, H., Massoud, Y., (eds). Extreme gradient boosting machine learning algorithm for safe auto insurance operations. In *2019 IEEE international conference on vehicular electronics and safety (ICVES)*; 2019: IEEE.

[42] Chen P, Yang Y, Zhang Y, Jiang S, Li X, Wan J (2020). Identification of prognostic immune-related genes in the tumor microenvironment of endometrial cancer. Aging.

[43] Song L, Wang X, Cheng W, Wu Y, Liu M, Liu R (2021). Expression signature, prognosis value and immune characteristics of cathepsin F in non-small cell lung cancer identified by bioinformatics assessment. BMC Pulm. Med..

[44] Liew SH, Nguyen Q-N, Strasser A, Findlay JK, Hutt KJ (2017). The ovarian reserve is depleted during puberty in a hormonally driven process dependent on the pro-apoptotic protein BMF. Cell Death Dis..

[45] Xu F, Xia T, Xu Q-T, Zhang X, Huang Y-Z, Sun X (2022). RBMS2 chemosensitizes breast cancer cells to doxorubicin by regulating BMF expression. Int. J. Biol. Sci..

[46] Badr EA, Assar MF, Eltorgoman AMA, Labeeb AZ, Breaka GA, Elkhouly EA (2020). A correlation between BCL-2 modifying factor, p53 and livin gene expressions in cancer colon patients. Biochem. Biophys. Rep..

[47] Guo X, Xiang C, Zhang Z, Zhang F, Xi T, Zheng L (2018). Displacement of Bax by BMF mediates STARD13 3′ UTR-induced breast cancer cells apoptosis in an miRNA-depedent manner. Mol. Pharm..

[48] Zheng X, Jia B, Lin X, Han J, Qiu X, Chu H (2016). FRMD4A: A potential therapeutic target for the treatment of tongue squamous cell carcinoma. Int. J. Mol. Med..

[49] Goldie SJ, Mulder KW, Tan DW-M, Lyons SK, Sims AH, Watt FM (2012). FRMD4A upregulation in human squamous cell carcinoma promotes tumor growth and metastasis and is associated with poor prognosis. Cancer Res..

[50] Pyfrom SC, Luo H, Payton JE (2019). PLAIDOH: A novel method for functional prediction of long non-coding RNAs identifies cancer-specific LncRNA activities. BMC Genomics.

[51] Zhang M, Wang X, Chen X, Guo F, Hong J (2020). Prognostic value of a stemness index-associated signature in primary lower-grade glioma. Front. Genet..

[52] Luo J, Wang W, Tang Y, Zhou D, Gao Y, Zhang Q (2017). mRNA and methylation profiling of radioresistant esophageal cancer cells: The involvement of Sall2 in acquired aggressive phenotypes. J. Cancer.

[53] Mosti F, Silver DL (2021). Uncovering the HARbingers of human brain evolution. Neuron.

[54] Liang J, Lv J, Liu Z (2015). Identification of stage-specific biomarkers in lung adenocarcinoma based on RNA-seq data. Tumor Biol..

[55] Girskis KM, Stergachis AB, DeGennaro EM, Doan RN, Qian X, Johnson MB (2021). Rewiring of human neurodevelopmental gene regulatory programs by human accelerated regions. Neuron.

[56] Thompson CH, Saxena A, Heelan N, Salatino J, Purcell EK (2021). Spatiotemporal patterns of gene expression around implanted silicon electrode arrays. J. Neural Eng..

[57] Liu P, Li Y, Zhang Y, Choi J, Zhang J, Shang G (2022). Calcium-related gene signatures may predict prognosis and level of immunosuppression in gliomas. Front. Oncol..

[58] Singh HN, Rajeswari MR (2016). Identification of genes containing expanded purine repeats in the human genome and their apparent protective role against cancer. J. Biomol. Struct. Dyn..

