# Supplementary material for: Down regulation of Cathepsin W is associated with poor prognosis in pancreatic cancer
Source: Sci Rep. 2023 Oct 4;13:16678. doi: 10.1038/s41598-023-42928-y (PMC10551021; doi:10.1038/s41598-023-42928-y)
Supplement: Supplementary file 1 — Supplementary Information. [file 41598_2023_42928_MOESM1_ESM.docx]

**
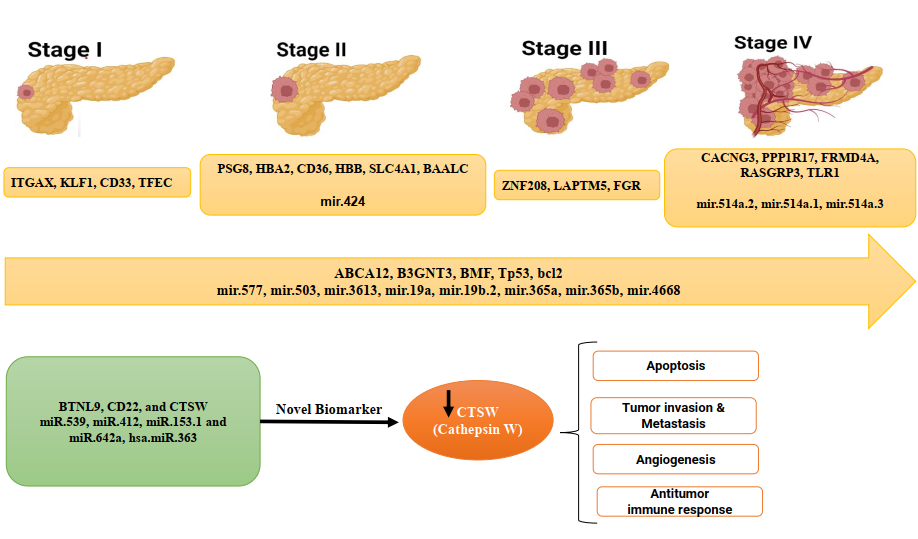
Supplement Figure**

**Supplement figure 1.** Schematic illustrates the involved genes and miRNAs  in the tumorigenesis of pancreatic

**Supplement Tables**

**Supplement table1:** Characteristics of population

| Characteristics | Subgroups | Frequency (%) |
| --- | --- | --- |
| Present of PC | Case | 185(95.9) |
|  | Control | 8(4.1) |
| Gender | Male | 107 (55.4) |
|  | Female | 86 (44.6) |
| Prior Malignancy | yes | 21 (10.9) |
|  | no | 172(89.1) |
| Alcohol Consumption | yes | 111 (57.5) |
|  | no | 82 (42.5) |
| Stages | 0 | 3 (1.6) |
|  | 1 | 21 (10.9) |
|  | 2 | 159 (82.4) |
|  | 3 | 3 (1.6) |
|  | 4 | 5 (3.6) |
| Status | alive | 88(45.5) |
|  | death | 105(54.5) |
|  | **Mean** | **SD** |
| Age | 64.59 | 11 |

**Supplement table 2.** Clinicopathological characteristics of the study groups, means ± SD or N (%).

| Characteristic | Patients (%) |
| --- | --- |
| Age (y) | 61.66±12.50 |
| Sex  Female  Male | 11 (52.4)  10 (47.6) |
| TMN classification  Stage I-II  Stage III-IV | 10 (47.6)  11 (52.4) |
| Tumor size  T1  T2  T3  T4 | 0 (0)  10 (47.6)  8 (38.1)  3 (14.3) |
| Nodal status  Yes  No | 14 (66.7)    7 (33.3) |
| Distant metastasis  Yes  No | 3 (14.3)  18 (85.7) |
| Grade  Poor-differentiated  Moderated-differentiated  Well-differentiated  Undifferentiated | 0 (0)  1 (4.8)  4 (19)  16 (76.2) |
